# Regeneration associated transcriptional signature of retinal microglia and macrophages

**DOI:** 10.1038/s41598-019-41298-8

**Published:** 2019-03-18

**Authors:** Diana M. Mitchell, Chi Sun, Samuel S. Hunter, Daniel D. New, Deborah L. Stenkamp

**Affiliations:** 1 Department of Biological Sciences, University of Idaho, Moscow, ID 83844 USA; 2 Present Address: Ophthalmology, Washington University in St. Louis, 4523 Clayton Ave St. Louis, Missouri, 63110 USA; 3 Institute for Bioinformatics and Evolutionary Studies, University of Idaho, Moscow, ID 83844 USA

## Abstract

Zebrafish have the remarkable capacity to regenerate retinal neurons following a variety of damage paradigms. Following initial tissue insult and a period of cell death, a proliferative phase ensues that generates neuronal progenitors, which ultimately regenerate damaged neurons. Recent work has revealed that Müller glia are the source of regenerated neurons in zebrafish. However, the roles of another important class of glia present in the retina, microglia, during this regenerative phase remain elusive. Here, we examine retinal tissue and perform QuantSeq. 3′mRNA sequencing/transcriptome analysis to reveal localization and putative functions, respectively, of *mpeg1* expressing cells (microglia/macrophages) during Müller glia-mediated regeneration, corresponding to a time of progenitor proliferation and production of new neurons. Our results indicate that in this regenerative state, *mpeg1*-expressing cells are located in regions containing regenerative Müller glia and are likely engaged in active vesicle trafficking. Further, *mpeg1*+ cells congregate at and around the optic nerve head. Our transcriptome analysis reveals several novel genes not previously described in microglia. This dataset represents the first report, to our knowledge, to use RNA sequencing to probe the microglial transcriptome in such context, and therefore provides a resource towards understanding microglia/macrophage function during successful retinal (and central nervous tissue) regeneration.

## Introduction

Microglia are resident immune cells of the macrophage lineage present in the vertebrate central nervous system (CNS). Recent work has identified crucial microglia-mediated functions in CNS development and maintenance^1–8^, substantially increasing our understanding of essential, intricate, and regulated interactions of microglia with neurons and with other glia in healthy CNS tissue. In contrast, in some contexts, microglia appear to contribute to pathology in neurodegenerative disease^9,10^. There is currently a lack of information regarding the function of microglia and microglia-derived factors, and that of any other immune cells present, in contexts of successful CNS (including retinal) regeneration, which occurs in non-mammalian vertebrates such as zebrafish. Further, limited markers currently exist to identify microglia and macrophages in zebrafish CNS tissue, although many reports support the zebrafish as a model for microglial biology^11–15^. Collectively, this limits our understanding of regenerative mechanisms, requirements for successful CNS regeneration, and regulation of an environment conducive to regeneration.

Our current understanding of retinal regenerative mechanisms is based largely upon the zebrafish model, and arises from the study of important roles of Müller glia (MG), rather than microglia. Following a variety of distinct damage paradigms resulting in death of retinal neurons in zebrafish, MG act as the source of new neurons in regenerated retinal tissues^16–21^. Gene expression and proteomic studies of retinal tissue during retinal regeneration in zebrafish have improved our understanding of this regenerative process^22–24^, but these studies did not differentiate between cell types contributing to transcript/protein abundance. Gene profiling studies of MG-derived progenitors have more specifically revealed transcriptional changes within this cell type in response to damage^25^, and have identified intrinsic factors required for regeneration shared by other regenerating tissues^26^. MG are also present in the mammalian retina; however, rather than regenerating neurons following injury or damage, a state of gliosis ensues^27–29^. Gliotic tissue is associated with hallmarks of inflammation, reactive microglia, and may result in glial scarring^27,28,30^. Interestingly, zebrafish mount a substantial microglia/macrophage response to a widespread retinal tissue lesion with inflammatory characteristics that is subsequently followed by successful neuronal regeneration^31^ and zebrafish reportedly display transient characteristics of gliosis^32,33^. Further, immune cell-MG crosstalk may shape MG reaction to retinal injury^34–36^, and possibly have effects on MG-mediated retinal regeneration^37^. Interestingly, a relatively recent study performed RNA sequencing of zebrafish MG-associated transcripts at early timepoints following light damage and found enrichment of genes categorized within cytokine signaling and immunity^25^. Recent reports have demonstrated FACS-based isolation of microglia and macrophage populations from other cell types in dissociated zebrafish CNS tissue for downstream RNA sequencing^12,38–40^. This has facilitated our understanding of the characteristics of zebrafish CNS microglia. However, such an analysis of microglia isolated from tissue engaged in a regenerative response in the CNS is necessary to better inform our understanding of microglia/macrophage contribution to CNS health and disease.

In zebrafish, a characteristic sequence of events follows retinal tissue injury. Upon death of retinal neurons, phagocytic cells (including microglia/macrophages and Müller glia) engulf debris arising from tissue insult^31,41,42^. A proliferative phase next ensues in which Müller glia re-enter the cell cycle and generate proliferative neuronal progenitors^16,18,19,21,31,43–45^. These progenitors then exit the cell cycle and differentiate to neurons resulting in regeneration of lost neuronal cell types^18,19,43,46,47^, and recovery of retinal tissue function^21,43,48^.

We previously reported that microglia and extra-retinally derived macrophages initially mount a robust response to widespread neuronal death induced by the neurotoxin ouabain^31^. This initial response is followed by a transition to MG proliferation^18,31^. Here, we investigate microglia/macrophage presence and characteristics during the subsequent phase involving MG-derived retinal regeneration. It is at this stage that zebrafish display a regenerative outcome, whereas mammals instead enter a phase corresponding to a gliotic response. Since microglia, and often other immune cells, participate in this gliotic response in mammals, we sought to reveal microglia/macrophage characteristics during active MG-mediated retinal regeneration in zebrafish. To this end, we examined localization of immune cells in regenerative retinal tissue and sequenced their mRNA transcriptome in this context. We find that immune cells present in regenerating tissue identify as microglia/macrophages based on expression of *mpeg1*-driven GFP^13,49^ and that microglia/macrophages in regenerating tissue show altered spatial distribution with congregations of microglia/macrophages localized to the optic nerve head and surrounding regions. To reveal the gene expression program of microglia/macrophages in such a context, we performed QuantSeq. 3′mRNA transcriptome analysis of *mpeg1*:GFP+ cells isolated from regenerating retinas and identified several novel genes not previously described in microglia, as well as a list of transcripts associated with *mpeg1*-expressing cells during retinal regeneration. We generated a list of candidate “regeneration-associated” transcripts, which showed enrichment of Gene Ontology categories suggesting increased vesicle trafficking within *mpeg1* expressing cells during retinal regeneration. This transcriptome data set provides a wealth of interesting and novel genes to be considered for follow-up studies towards identifying microglia/macrophage function during zebrafish retinal regeneration.

## Results

### Features of immune cells and Müller glia in regenerating retinal tissue

Recent studies have begun to reveal characteristics of microglia, including observations of their identity and features in retinal tissues, during MG reactivity and resulting retinal regeneration in zebrafish following neuronal damage^31,37^. To build on this foundation, we visualized localization and characteristics of immune cells (including microglia) in retinal tissue undergoing active regeneration following a tissue-disrupting lesion. We analyzed cryosections at seven days following intravitreal injection of a final concentration 2 μM of ouabain (7 dpi). This lesioning strategy has been shown to destroy inner retinal neurons, but to spare photoreceptors and MG^18,21,31,48^. The 7 dpi timepoint follows the initial response to tissue injury (which peaks approximately 1–2 dpi^18,31^) as well as the shift to the proliferative phase in which MG have re-entered the cell cycle (approximately 3 dpi). By 5 dpi, neuronal progenitors are detected^18^ and by 7 dpi, MG-derived progenitors begin to enter the regenerative phase^18,19^ as evidenced by detection of ganglion cell markers^18,21^, as well as markers of ganglion cell axon outgrowth^18^.

**Figure 1:**
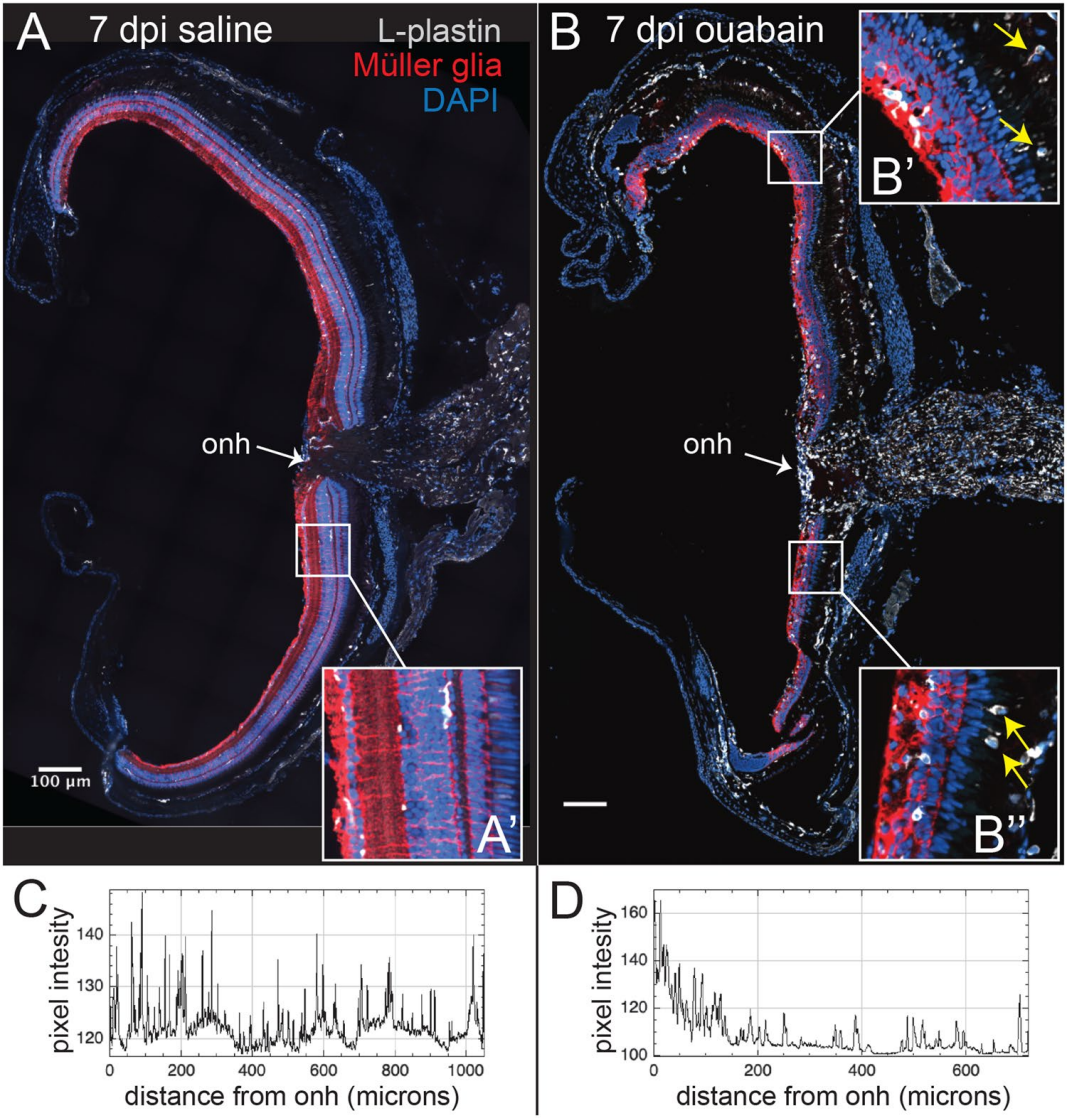
Immune cell features and distribution in regenerating retinal tissue. Images show retinal cryosections at 7 days post injection (7 dpi) of saline (**A**) or 2 μM final concentration of ouabain (**B**) stained for L-plastin (gray; microglia/macrophages), Glutamine Synthetase (GS, red; Müller glia), and DAPI (blue; nuclei). A and B show stitched images of entire cryosections, insets (A’, B’, and B”) show indicated enlarged regions. Müller glia in retinas 7 dpi ouabain display hypertrophy throughout the regenerating inner retina and appear disorganized (B’,B”) compared to control (A’). (**C**,**D**). Plots show pixel intensity of L-plastin+ signal as a distance from the optic nerve head (onh). L-plastin+ cells in saline injected retinas show even distribution and are ramified (A, A’,C), while L-plastin+ cells in regenerating retinas (B-B”) appear irregularly dispersed (**D**) and display ameboid morphology. B’ and B” reveal that the L-plastin+ cells in the inner retina conform to the network of Müller glial cells labeled by GS expression. In addition, L-plastin+ cells are densely localized in regions corresponding to the optic nerve head (onh) at 7 dpi ouabain, and several immune cells appear in regions apical to the retina with directional orientation that could suggest migration into retinal tissue from the RPE or outside of the retina (yellow arrows, B’ and B”). Scale bars in (**A**,**B**) = 100 μm.

To visualize microglial, and any other immune cell, features in this regenerative state, we used an antibody to L-plastin, which marks all immune cells including microglia^31,50,51^, and an antibody to glutamine synthetase (GS) to label MG. We observed that L-plastin+ cells were present within regenerating retinal tissue containing reactive GS-labeled MG within regions of the inner retina corresponding to the location of the initial retinal lesion (Fig. 1B,B’,B”). At 7 dpi, L-plastin+ cells appeared predominantly localized to this damage-specific region within the inner retina (Fig. 1B). Müller glia displayed hypertrophy (Fig. 1B,B’,B”, compared to Fig. 1A,A’), consistent with previous observations following a variety of damage paradigms^18,21,32^.

**Figure 2:**
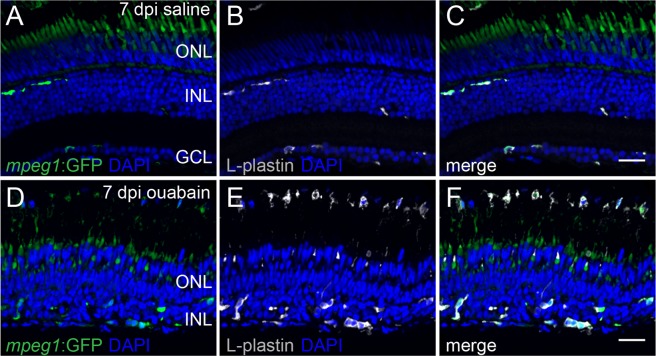
L-plastin+ cells in regenerating retinal tissue co-label with *mpeg1*-driven GFP. Images of a retinal cryosection at 7 dpi saline (**A**–**C**) or ouabain (**D**–**F**) injection obtained from *mpeg1*:GFP transgenic fish and visualized for *mpeg1*:GFP (green, **A**,**D**), L-plastin immunolabeling (gray, **B**,**E**), and DAPI staining (blue; nuclei, **A**–**F**). (**C**,**F**) Three color merge. L-plastin+ cells co-label with the *mpeg1*:GFP transgene. Autofluorescence from photoreceptors is visible in the outer nuclear layer (ONL) in other channels (**A**,**C**,**D**,**F**) in fixed cryosection images, however, this autofluorescence did not affect FACS sorting of GFP+ and GFP− populations from freshly isolated, dissociated regenerating retinas. Scale bar in (**C**,**F**) = 20 μm apply to (**A**–**F**). ONL: outer nuclear layer, INL: inner nuclear layer, GCL: ganglion cell layer.

**Figure 3:**
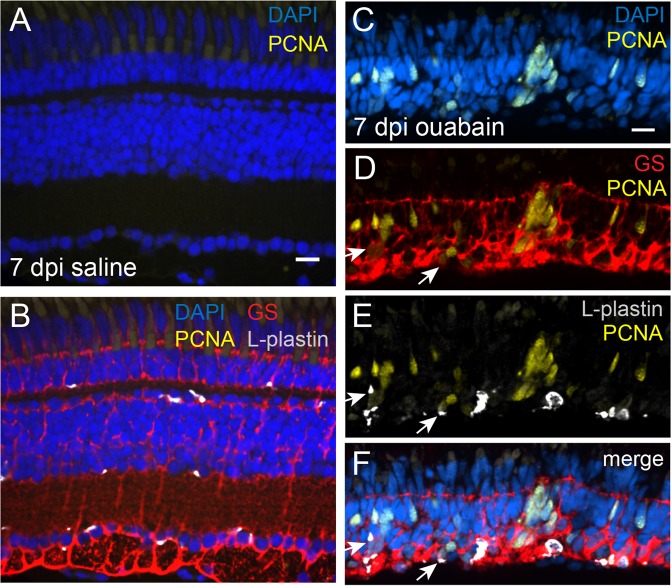
PCNA expression in regenerating retinal tissue at 7 days post ouabain injection. Images of a retinal cryosection at 7 dpi saline (**A**,**B**) or ouabain injection (**C**–**F**) stained for DAPI (blue; nuclei), PCNA (yellow), Glutamine Synthetase to mark Müller glia and Müller glia-derived progenitors (red), and L-plastin to mark immune cells (gray). (**A**,**C**) DAPI and PCNA stain. (**D**) GS and PCNA stain. (**E**) L-plastin and PCNA stain. (**B**,**F**) Four color merge. In regenerating retinal tissue, cells expressing PCNA cels are visible throughout the inner retina, corresponding to regions of initial tissue damage, and often appear in clusters. Most PCNA signal can be attributed to cytoplasm containing GS signal. Occasionally, PCNA signal is associated with L-plastin+ cytoplasm (**E**), but this often overlaps with GS signal (arrows, **D**–**F**). Scale bar in A = 10 μm, applies to (**A**,**B**). Scale bar in C = 10 μm, applies to (**C**–**F**).

L-plastin+ cells in regenerating retinas were irregularly distributed throughout the retinal tissue (Fig. 1B,D), rather than showing a more evenly spaced distribution as in control retinas (Fig. 1A,C), and in contrast to that seen in acutely damaged ouabain injected retinas^31^. L-plastin+ cells within the regenerating retina appeared to predominantly localize to the basal portions of the regenerating inner retina, while only rarely seen to be localized to apical regions corresponding to Müller glia-derived neuronal progenitor proliferation^19,52^ (Figs 1–3). The L-plastin+ cells were not ramified and instead were irregular in shape (Figs 1B,B’,B” and 2D–F).

We also observe that L-plastin+ cells appeared most densely present in regions corresponding to, and surrounding, the optic nerve head (onh, Fig. 1B,D) in regenerating retinas, and density of L-plastin+ cells was reduced in a gradient as a function of distance from the onh (Fig. 1A–D). Further, many L-plastin+ cells were visible in tissues apical to the retina and were directionally (radially) oriented towards retinal tissue (Fig. 1B’,B”, arrows). It is possible that these apically positioned L-plastin+ cells may represent RPE-associated microglia that migrate into the regenerating retina, or possibly extra-retinally derived immune cells that continue to infiltrate the retina beyond the previously documented timepoint of 3 dpi^31^. L-plastin+ cells within retinal tissue at 7 dpi ouabain or saline co-labeled with the macrophage-specific *mpeg1*-driven GFP transgene (Fig. 2), therefore indicating that these immune cells consist of microglia^13^ and possibly other infiltrating macrophages.

Since 7 dpi corresponds to near-peak numbers of cells expressing PCNA^18^, we stained and imaged cryosections of regenerating retina to visualize PCNA in combination with L-plastin and GS (Fig. 3) to determine if any L-plastin+ cells express PCNA. PCNA signal was not detected in tissue at 7 dpi following saline injection (Fig. 3A,B). As previously reported^18^, PCNA signal was detected throughout the inner retina at 7 dpi following ouabain injection, often found in clusters, and nuclei with PCNA signal adopted an elongated shape (Fig. 3C–F). We observe that most of the nuclei containing PCNA at 7 dpi ouabain were associated with GS-labeled cytoplasmic signal (Fig. 3D). Occasionally, PCNA signal was associated with L-plastin+ cytoplasm, but this often overlapped with GS signal (Fig. 3D–F, arrows), making it difficult to attribute proliferation to MG (GS+) versus L-plastin+ cells. Collectively, this indicates that PCNA expression at this timepoint primarily represents reactive MG and MG-derived progenitors.

### Transcriptome analysis of *mpeg1*:GFP+ cells during retinal regeneration

**Figure 4:**
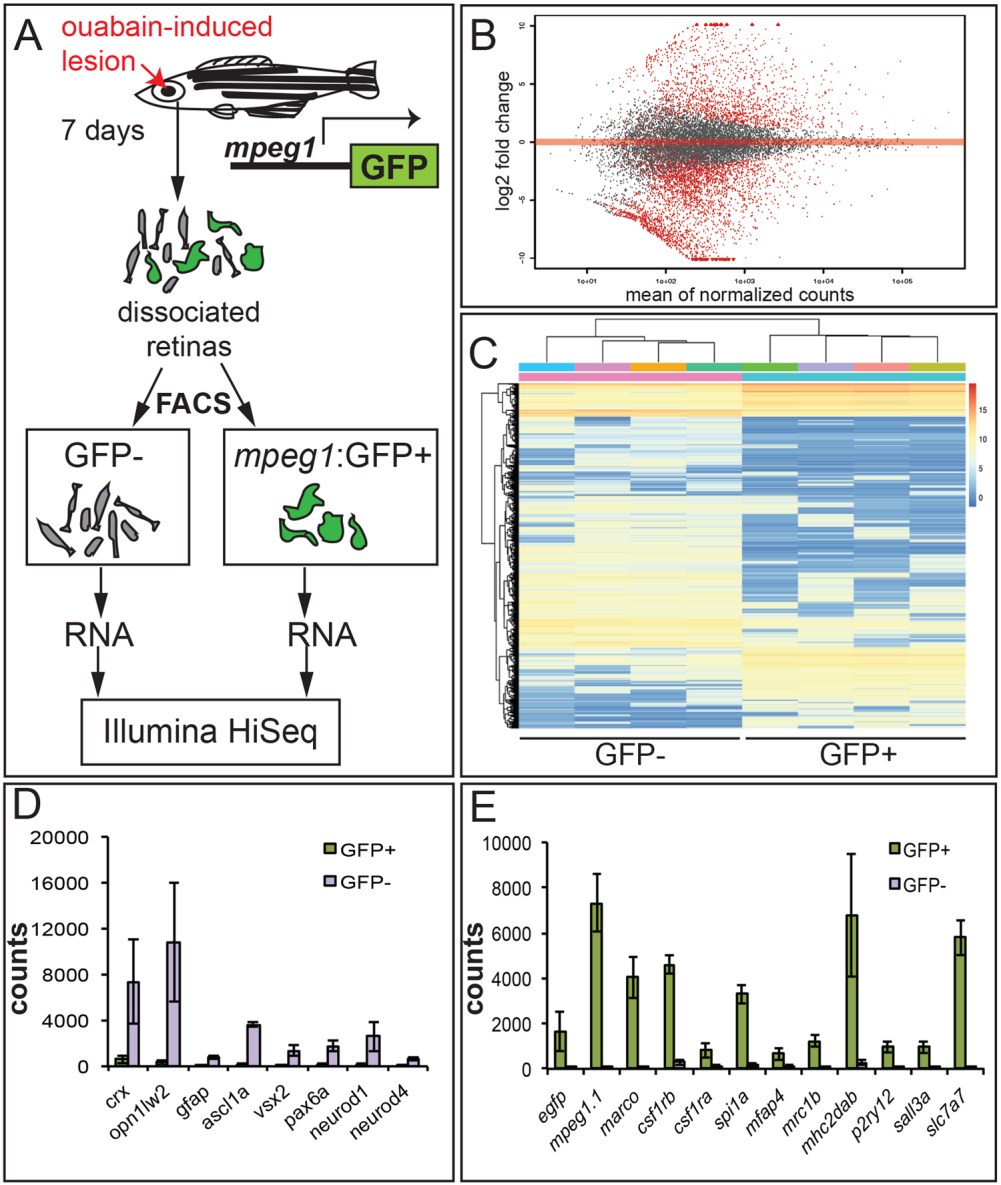
Overview of RNA-sequencing of *mpeg1*:GFP+ and GFP− populations isolated from regenerating retinas. (**A**) At 7 days post ouabain lesion (7 dpi), retinas from *mpeg1*:GFP+ fish were dissociated and subjected to Fluorescence Activated Cell Sorting (FACS) to obtain purified populations of GFP+ and GFP− cells, RNA was isolated from respective populations, and then sequenced by Illumina HiSeq. (**B**) MA plot of moderated Log2 fold change obtained from RNA sequencing. (**C**) Heat map showing row centered rlog transformed values for all genes differentially expressed with p < 0.1, to illustrate expression relative to each (GFP− or GFP+) population. The legend in upper right indicates transcript abundance relative to the other population. (**D**) Raw counts of sequences mapped to genes associated with photoreceptors (*crx*, *opn1lw2*, which survive the retinal lesion), and Müller glia and neuronal progenitors (*gfap*, *ascl1a*, *vsx2*, *pax6a*, *neurod1*, *neurod2*) in GFP+ and GFP− samples. E. Raw counts of sequences mapped to genes associated with *egfp*, macrophages (*mpeg1*.*1*, *marco*, *csf1a*, *csf1b*, *spi1a*, *mfap4*, *mrc1b*, *mhc2dab*), and microglia (*p2ry12*, *sall3a*, *slc7a7*) in GFP+ and GFP− populations. Error bars in (**D**,**E**) represent SEM.

Since functions of immune cells, including microglia, in the context of successful retinal (or CNS) regeneration are not well understood, and to build on previous gene expression studies during zebrafish retinal regeneration^22,23,25^, we sought to identify the microglia (and macrophage)-specific transcriptome corresponding to an environment of successful regeneration, to gain insight into those possible functions. As described above, L-plastin+ cells localized within retinal tissue obtained from *mpeg1*:GFP transgenic zebrafish at 7 dpi co-labeled with *mpeg1*-driven GFP (Fig. 2). *Mpeg1*-driven GFP expression allowed us the opportunity to use GFP signal as a basis to isolate GFP+ microglia/macrophages^13,49^ from other retinal cell types (GFP−) during active retinal regeneration, followed by QuantSeq. 3′-mRNA sequencing, to reveal their respective transcriptomes (Fig. 4).

We used Fluorescence Activated Cell Sorting (FACS) to isolate highly pure populations of GFP+ and GFP− cells from dissociated cell suspensions from *mpeg1*:GFP transgenic retinas at 7 days post ouabain lesion followed by RNA isolation^38^ and (Fig. 4). The results of this dissociation, cell sorting, confirmation of purity, and RNA quality were reported in^38^. To identify the mRNA transcripts present in *mpeg1*:GFP+ cells compared to GFP− cells during zebrafish retinal regeneration, we performed QuantSeq. 3′-mRNA sequencing of RNA isolated from the sorted GFP+ and GFP− cells. Sequencing of GFP− cells was also included in this experiment in order to compare transcript expression in non-microglial/macrophage populations at this timepoint. Comparison of transcripts between such populations is useful because very little is known about the zebrafish microglia/macrophage transcriptome, and such insight will increase our understanding of these cell types in the zebrafish model organism. Using criteria of moderated log2FC > |2| and FDR < 0.01, we identified 2779 differentially expressed genes, with 970 of these significantly enriched in GFP+ cells (Fig. 4B, Table 1 and Supplementary File S2) and 1809 of these significantly enriched in GFP− cells (Fig. 4B, Table 2 and Supplementary File S2). Principal Component Analysis showed that the sorted GFP+ and GFP− populations formed two highly distinct clusters, indicating that these populations contain distinct transcripts representing different gene expression programs (Supplemental Fig. S1). A heat map was generated based on rlog transformed values of differentially expressed genes with p < 0.1 to broadly illustrate differential expression of transcripts relative to each population (Fig. 4C). This heat map reveals a minor cluster of genes with similar expression levels between GFP+ and GFP− populations, as well as clusters of genes with relatively different expression levels, and reveals only slight variation among samples within groups.

**Table 1:** Top 50 transcripts enriched in *mpeg1*:GFP+ cells compared to GFP− retinal cells at 7 days post ouabain lesion, sorted by FDR.

moderated log2FC	FDR	GENE NAME	SYMBOL	ZFIN	ENTREZID
6.947697132	8.29E-48	apolipoprotein C-I	apoc1	ZDB-GENE-030131-1074	570638
6.935549678	1.21E-38	solute carrier family 43, member 3b	slc43a3b	ZDB-GENE-060312-28	556206
9.744245899	2.75E-37	regulator of G protein signaling 18	rgs18	ZDB-GENE-061013-722	768164
8.364686368	9.84E-37	chemokine (C-C motif) ligand 34b, duplicate 1	ccl34b.1	ZDB-GENE-091204-276	563952
9.292757461	1.03E-36	hepatitis A virus cellular receptor 1	havcr1	ZDB-GENE-040718-131	436707
5.714666395	2.75E-36	lectin, galactoside-binding, soluble, 9 (galectin 9)-like 1	lgals9l1	ZDB-GENE-030131-9543	337597
10.82966572	4.22E-34	uncharacterized LOC100150882	LOC100150882	NA	100150882
6.712331301	6.09E-32	CD74 molecule, major histocompatibility complex, class II invariant chain b	cd74b	ZDB-GENE-990910-10	30645
5.887950241	4.26E-30	CD74 molecule, major histocompatibility complex, class II invariant chain a	cd74a	ZDB-GENE-000901-1	58113
8.897462506	2.20E-29	solute carrier family 7 (amino acid transporter light chain, y+ L system), member 7	slc7a7	ZDB-GENE-051127-5	641560
6.139928731	1.38E-28	si:busm1-266f07.2	si:busm1-266f07.2	ZDB-GENE-030616-436	368878
8.549659679	6.09E-27	interleukin 10 receptor, alpha	il10ra	ZDB-GENE-070905-4	777651
8.850007997	6.13E-26	integrin, alpha E, tandem duplicate 1	itgae.1	ZDB-GENE-131121-125	100333951
8.699101471	1.84E-25	si:ch73-203a8.1	si:ch73-203a8.1	ZDB-GENE-070912-335	100151151
10.15171232	1.04E-24	protein NLRC3-like	LOC100334101	NA	100334101
8.913425562	1.79E-24	interleukin-1 receptor type 2	LOC101882195	NA	101882195
5.61985783	1.81E-24	complement component 1, q subcomponent, B chain	c1qb	ZDB-GENE-040801-223	445088
6.670581809	3.36E-24	G0/G1 switch 2	g0s2	ZDB-GENE-081022-103	568476
6.506472448	5.92E-24	retinol binding protein 2a, cellular	rbp2a	ZDB-GENE-020320-2	568032
4.928284874	1.57E-23	profilin 1	pfn1	ZDB-GENE-031002-33	799355
6.183866184	2.84E-23	lymphocyte cytosolic protein 1 (L-plastin)	lcp1	ZDB-GENE-991213-5	30583
8.504782713	7.69E-22	macrophage receptor with collagenous structure	marco	ZDB-GENE-120514-2	571584
6.059783514	7.69E-22	granulin 1	grn1	ZDB-GENE-060103-1	553977
8.067781947	7.85E-22	macrophage expressed 1, tandem duplicate 1	mpeg1.1	ZDB-GENE-030131-7347	335407
4.183893764	9.14E-22	CCAAT/enhancer binding protein (C/EBP), beta	cebpb	ZDB-GENE-020111-3	140814
9.25435495	6.52E-21	si:ch211-102c2.4	si:ch211-102c2.4	ZDB-GENE-030131-8862	568178
4.122049372	8.44E-21	fatty acid binding protein 11a	fabp11a	ZDB-GENE-040912-132	447944
4.958997926	1.18E-20	coronin, actin binding protein, 1 A	coro1a	ZDB-GENE-030131-9512	100002113
5.179139762	2.01E-20	Fc receptor, IgE, high affinity I, gamma polypeptide like	fcer1gl	ZDB-GENE-070502-4	100101653
7.846346992	3.41E-20	connexin 32.2	cx32.2	ZDB-GENE-050303-1	566647
4.234645883	4.88E-20	cathepsin C	ctsc	ZDB-GENE-030619-9	368704
4.507055275	5.17E-20	legumain	lgmn	ZDB-GENE-021030-1	406625
8.734468946	7.90E-20	GRB2-related adaptor protein a	grapa	ZDB-GENE-050522-347	553596
8.9405533	1.04E-19	interleukin-1 family member A	il1fma	ZDB-GENE-140106-231	102997062
6.437918568	1.35E-19	lectin, galactoside-binding, soluble, 3 binding protein b	lgals3bpb	ZDB-GENE-040426-2262	405809
8.410056881	1.51E-19	neutrophil cytosolic factor 1	ncf1	ZDB-GENE-031006-6	378966
7.080422693	2.03E-19	apoptosis facilitator Bcl-2-like protein 14	LOC101885512	NA	101885512
6.427189127	3.22E-19	chemokine (C-X-C motif) ligand 19	cxcl19	ZDB-GENE-140708-2	100003911
6.677182232	4.15E-19	integrin, beta 2	itgb2	ZDB-GENE-110411-4	557797
8.305047042	5.60E-19	si:dkey-88j15.3	si:dkey-88j15.3	ZDB-GENE-100922-28	560795
4.407928906	7.81E-19	GM2 ganglioside activator	gm2a	ZDB-GENE-050417-373	550529
4.341624501	1.03E-18	Rho GDP dissociation inhibitor (GDI) gamma	arhgdig	ZDB-GENE-040426-1493	394132
7.971312316	1.07E-18	si:cabz01074946.1	si:cabz01074946.1	ZDB-GENE-160113-134	100331505
7.338888853	1.15E-18	lysosomal-associated protein transmembrane 5	LOC100151049	NA	100151049
7.269771345	1.15E-18	protein tyrosine phosphatase, non-receptor type 6	ptpn6	ZDB-GENE-030131-7513	335573
9.665266832	2.03E-18	cytotoxic and regulatory T-cell molecule	crtam	NA	101884219
6.67300911	2.46E-18	tumor necrosis factor b (TNF superfamily, member 2)	tnfb	ZDB-GENE-050601-2	554167
5.681315201	1.12E-17	glutathione S-transferase omega 2	gsto2	ZDB-GENE-041114-67	492500
4.081012634	1.44E-17	sequestosome 1	sqstm1	ZDB-GENE-040426-2204	406452
6.184453342	1.62E-17	IL-6 subfamily cytokine M17	m17	ZDB-GENE-060526-368	555717

**Table 2:** Top 50 transcripts depleted in *mpeg1*:GFP+cells compared to GFP− retinal cells at 7 days post ouabain lesion, sorted by FDR.

moderated log2FC	FDR	GENE NAME	SYMBOL	ZFIN	ENTREZID
−9.902497402	4.52E-39	prostaglandin D2 synthase b, tandem duplicate 2	ptgdsb.2	ZDB-GENE-030911-3	751701
−5.354755121	1.18E-29	arrestin 3a, retinal (X-arrestin)	arr3a	ZDB-GENE-040718-102	436678
−8.788432384	4.96E-27	biglycan b	bgnb	ZDB-GENE-040426-21	792197
−5.457182226	3.46E-24	phosphodiesterase 6 H, cGMP-specific, cone, gamma, paralog a	pde6ha	ZDB-GENE-040426-1754	393758
−5.100382872	1.57E-23	guanine nucleotide binding protein (G protein), beta polypeptide 3b	gnb3b	ZDB-GENE-040426-2280	406483
−9.975719622	1.35E-22	ba1 globin	ba1	ZDB-GENE-990415-18	30216
−5.337784049	1.35E-22	recoverin 2	rcvrn2	ZDB-GENE-030131-7590	335650
−6.435756586	1.46E-22	guanine nucleotide binding protein (G protein), gamma transducing activity polypeptide 2b	gngt2b	ZDB-GENE-091020-4	797361
−6.397569387	8.07E-22	opsin 1 (cone pigments), short-wave-sensitive 1	opn1sw1	ZDB-GENE-991109-25	30582
−6.09923235	8.44E-21	phosphodiesterase 6 C, cGMP-specific, cone, alpha prime	pde6c	ZDB-GENE-040426-1664	393845
−8.761307281	1.03E-19	ral guanine nucleotide dissociation stimulator-like 3a	rgl3a	ZDB-GENE-101130-1	100149956
−7.8244016	1.77E-19	MYCN proto-oncogene, bHLH transcription factor	mycn	ZDB-GENE-020711-1	252851
−6.044518098	2.44E-19	insulin-like growth factor binding protein 5b	igfbp5b	ZDB-GENE-040319-2	403039
−8.029031786	3.45E-19	solute carrier family 38, member 4	slc38a4	ZDB-GENE-041010-14	449771
−5.288624547	3.45E-19	aquaporin 1a (Colton blood group), tandem duplicate 1	aqp1a.1	ZDB-GENE-030131-7764	335821
−5.836694213	6.71E-19	guanine nucleotide binding protein (G protein), gamma transducing activity polypeptide 2a	gngt2a	ZDB-GENE-030131-7595	335655
−7.439163089	1.13E-18	NA	NA	NA	NA
−4.079039413	1.45E-18	fatty acid binding protein 7, brain, a	fabp7a	ZDB-GENE-000627-1	58128
−6.971250125	3.09E-18	solute carrier family 24 (sodium/potassium/calcium exchanger), member 2	slc24a2	ZDB-GENE-060825-277	751686
−8.382635563	6.75E-18	unc-119 homolog b (C. elegans)	unc119b	ZDB-GENE-050201-2	678653
−8.363908429	1.26E-17	follistatin b	fstb	ZDB-GENE-031118-139	566538
−8.099791471	1.44E-17	espin	espn	ZDB-GENE-081105-173	567061
−7.466759677	4.39E-17	fatty acid desaturase 2	fads2	ZDB-GENE-011212-1	140615
−4.673096962	6.59E-17	retinol binding protein 4, like	rbp4l	ZDB-GENE-030131-7591	335651
−6.617741567	1.62E-16	synaptotagmin Va	syt5a	ZDB-GENE-040718-110	436686
−6.853925862	1.83E-16	peripherin 2a (retinal degeneration, slow)	prph2a	ZDB-GENE-000616-8	58085
−4.326891	3.67E-16	midkine a	mdka	ZDB-GENE-990621-1	30277
−10.78175227	4.55E-16	T-box 2b	tbx2b	ZDB-GENE-990726-27	30253
−10.29982042	5.43E-16	si:ch211-183d21.1	si:ch211-183d21.1	ZDB-GENE-030131-8516	571430
−7.361502097	5.82E-16	doublecortin-like kinase 2a	dclk2a	ZDB-GENE-050420-170	572548
−10.57382742	6.16E-16	protein kinase C and casein kinase substrate in neurons 1a	pacsin1a	ZDB-GENE-050522-155	553559
−6.109615504	6.38E-16	zgc:195173	zgc:195173	ZDB-GENE-081022-190	792613
−10.29473981	7.27E-16	transmembrane protein 108	tmem108	ZDB-GENE-091204-397	563089
−5.473704873	1.73E-15	opsin 1 (cone pigments), long-wave-sensitive, 2	opn1lw2	ZDB-GENE-040718-141	436716
−7.717505976	2.51E-15	tight junction protein 2b (zona occludens 2)	tjp2b	ZDB-GENE-040718-58	436639
−8.088389566	5.77E-15	retinol binding protein 3	rbp3	ZDB-GENE-990415-132	30735
−8.358797824	6.92E-15	storkhead box 2a	stox2a	ZDB-GENE-090313-101	571741
−5.691417183	1.38E-14	ATPase, Na+/K+ transporting, alpha 1b polypeptide	atp1a1b	ZDB-GENE-001212-5	64616
−6.468437477	1.87E-14	collagen type XVIII alpha 1 chain a	col18a1a	ZDB-GENE-030516-3	564123
−5.409702873	2.17E-14	solute carrier family 1 (glial high affinity glutamate transporter), member 2b	slc1a2b	ZDB-GENE-030131-7779	335836
−8.857169039	2.50E-14	erythrocyte membrane protein band 4.1 like 5	epb41l5	ZDB-GENE-030616-450	368449
−7.392218598	3.00E-14	si:ch211-81a5.8	si:ch211-81a5.8	ZDB-GENE-060503-138	560648
−9.889739986	4.98E-14	BOC cell adhesion associated, oncogene regulated	boc	ZDB-GENE-030131-6560	334628
−9.997009725	5.09E-14	hairy-related 2	her2	ZDB-GENE-980526-274	30300
−7.35634514	5.33E-14	si:dkey-126g1.9	si:dkey-126g1.9	ZDB-GENE-030131-9862	792999
−10.86276442	9.44E-14	collagen, type V, alpha 2a	col5a2a	ZDB-GENE-030616-13	564821
−8.375593909	9.47E-14	shroom family member 2a	shroom2a	ZDB-GENE-050208-128	386817
−7.42402034	1.04E-13	NA	NA	NA	NA
−4.477364135	1.08E-13	ATPase, H+ transporting, lysosomal, V0 subunit cb	atp6v0cb	ZDB-GENE-030131-4127	325402
−10.42654367	1.69E-13	si:ch211-285j22.3	si:ch211-285j22.3	ZDB-GENE-141216-187	336034

To confirm the output of this design, we probed our data set for selected transcripts in GFP+ and GFP− populations that represent microglia/macrophages and other retinal cell types, respectively. Transcripts pertaining to genes expressed by the monocyte (*spi1a*, *csf1ra*, *csf1rb*^53–55^) and macrophage lineages *(mhc2dab*, *marco*, and *mfap4*^56,57^), and transcripts expressed by microglia (*p2ry12*, *sall3a*, *slc7a7*^58–63^), were significantly enriched in GFP+ samples based on Differential Expression and raw counts (Fig. 4E, Table 1, and Supplementary File S1). Importantly, GFP+ populations were enriched for the macrophage/microglia-specific gene *mpeg1*.*1* as well as *egfp* transcripts, which formed the basis of our FACS sorting (Fig. 4E and Table 1). On the other hand, genes expressed by photoreceptors, including those involved in photoreceptor identity and function (e.g. *crx*, *opn1lw2*, *arr3a*, *rcvrn2*, *gngt2a*, *syt5a*^64–70^), were highly enriched in the GFP− population and only minimally detected in the GFP+ population (Fig. 4D, Table 3, and Supplementary File S1), consistent with previous reports that photoreceptors survive the inner retina-selective ouabain lesion^18,21,31,48^. Genes expressed by neuronal progenitors (e.g. *vsx2*, *neurod1*, *neurod4*, and *pax6a*^33^) were also highly enriched in the GFP− population (Fig. 4D, Table 2, and Supplementary File S2), consistent with engagement of a regenerative state. Müller glia-associated *gfap* expression was also significantly enriched in GFP− cells, as well as other genes upregulated in Müller glia during zebrafish retinal regeneration (such as *mdka* and *ascl1a*^71–74^) (Fig. 4D, Table 2, Supplementary File S2). In addition, several members of the Notch family (e.g. *notch1b*, *notch3*, *jag1b*, *dla*) were enriched in GFP− populations, consistent with a role for Notch signaling in regeneration of retinal neurons in zebrafish^75–77^ (Table 2, Supplementary File S2).

**Table 3:** qPCR validation of selected transcripts found to be enriched in GFP+ compared to GFP− populations.

		qPCR transcript detection, Ct values		Fold expression, qPCR
Transcript	DE, RNA-seq	*mpeg1*:GFP+	GFP−	
*lcp1*	+6.18	29.36	unreliable^§^	N/A
*mpeg1*.*1*	+8.07	29.90	unreliable	N/A
*p2ry12*	+8.69	27.17	30.61	67.87
*itgb2*	+6.68	29.60	33.10	45.08
*apoc1*	+6.95	23.99	25.80	30.29
*c1qa*	+5.84	28.40	31.50	35.01
*c1qb*	+5.62	32.40	unreliable	N/A
*cfp*	+4.03	30.06	unreliable	N/A
*il1b*	+5.53	31.20	unreliable	N/A
*lgals3bpb*	+6.45	22.73	27.39	102.98
*18 s*		26.83	24.86	

^§^Unreliable: Average Ct value ≥ 34 (indicating single copy levels) and/or Ct value reported as “undetermined” by the instrument for at least 2 of 3 samples.

N/A: Not applicable.

Several selected transcripts identified in the RNA-seq experiment as differentially expressed in GFP+ microglia/macrophages were confirmed by qPCR analysis of GFP+ and GFP− populations sorted from *mpeg1*:GFP+ retinas at 7 dpi in a separate experiment (Tables 3 and 4). Transcripts associated with leukocytes and macrophages (*lcp1* and *mpeg1*.*1*, respectively^49,50^, previously reported to be selectively amplified in the GFP+ sorted population subject to RNA-seq^38^) and microglia (*p2ry12*^62^) were confirmed to be enriched in GFP+ populations compared to GFP− populations (Table 3). We also confirmed that several complement factors identified in the RNA-seq (*c1qa*, *c1qb*, *cfp*, and the complement receptor subunit *itgb2*) were selectively amplified from GFP+ populations (Table 3). In addition, as indicated by the RNA-seq experiment, we also found *apoc1*, *il1b*, and *lgals3bpb* to be enriched in *mpeg1*:GFP+ cells compared to GFP− cells by qPCR (Table 3).

**Table 4:** qPCR validation of selected transcripts found to be depleted in GFP+ compared to GFP− populations.

		qPCR transcript detection, Ct values		Fold expression, qPCR
Transcript	DE, RNA-seq	*mpeg1*:GFP+	GFP−	
*crx*	−3.82	31.10	27.19	0.30
*opn1lw2*	−5.47	not detected^‡^	22.24	N/A
*gfap*	−4.70	unreliable^§^	31.60	N/A
*pax6a*	−4.24	unreliable	30.33	N/A
*ascl1a*	−4.59	not detected	32.12	N/A
*mef2ca*	−3.80	not detected	30.76	N/A
*ncam1b*	−2.82	30.58	26.82	0.60
*sept8b*	−8.97	33.30	28.81	0.19
*pdca*	−6.92	not detected	26.70	N/A
*prom1b*	−7.25	not detected	29.41	N/A
*18 s*		26.83	24.86	

^**‡**^Not detected: Ct value reported as “undetermined” by the instrument for all samples.

^§^Unreliable: Average Ct value ≥ 34 (indicating single copy levels) and/or Ct value reported as “undetermined” by the instrument for at least 2 of 3 samples.

N/A: Not applicable.

Further consistent with our RNA-seq experiment, transcripts associated with photoreceptors (*crx*, *opn1lw2*) were confirmed to be enriched in GFP− populations using qPCR (Table 4), consistent with the survival of photoreceptors in this damage system^18,21,31,48^. Transcripts expressed by Müller glia and Müller glia-derived progenitors during retinal regeneration (*gfap*, *pax6a*, *ascl1a*^20,74^) were confirmed as enriched in the GFP− population by qPCR analysis (Table 5). Several other transcripts (*mef2ca*, *ncam1b*, *sept8b*, *pdca*, *prom1b*) found to be enriched in GFP− compared to GFP+ cells in the RNA-seq experiment showed a similar result when analyzed by qPCR (Table 4).

**Table 5:** Primer sequences used for qPCR.

Gene	Sense (forward) 5′→3′	Anti-sense (reverse) 5′→3′
*lcp1*	GCAGTGGGTGAACGAAACAC	TCGAGATCGCATACTTGGCG
*mpeg1*.*1*	CATGTCGTGGCTGGAACAGA	ATGGTTACGGACTTGAACCCG
*p2ry12*	AGCGTCTCCAACAGTTCATCC	GCCAGAGCGTTCAGGGATAATC
*itgb2*	TGCTGGTAAAGACCCAGTGC	TTTGGGGCATCCCTGGTCTA
*c1qa*	TGACAGCGAGACACTGATGTT	GCGCCATTTCTTCCATGCTT
*c1qb*	GATCCAGGTGAGAATGCAGTG	TCCCTCTGGTCCCTTCACAC
*apoc1*	AAGACCAAAACCGCCTTCCA	GGGGGTGTAAGGTAAATGGGG
*il1b*	TTCCCCAAGTGCTGCTTATT	AAGTTAAAACCGCTGTGGTCA
*cfp*	TCCTCAGCCTGCTCTGTGACTTGTG	TTCGGGTTCCCTCCCTGGTTCT
*lgals3bpb*	GGTGGACATCAGCCAGACTT	CCAGCTGATGGAGACACTGA
*crx*	CATAACTGGAGGGGAATCTG	AAAGCACGACACAAGAACTC
*pax6a*	CACATACACACCCCCGCAC	CCGAGGCGGCCATTG
*ascl1a*	GCCAGACGGAACGAGAGAGA	AGGGTTGCAAAGCCGTTG
*gfap*	CTAAGCCAGACTTGACCGCT	TTACGATTGGCTGCATCCGT
*opn1lw2*	AGAGGGAAGAACTGGACTTTCAGA	TTCAGAGGAGTTTTGCCTACATATGT
*mef2ca*	TGTAATCATTCAGCGTAGTG	TCTAAGGTGTGCCGTTAT
*ncam1b*	AGTTTGATAAAGATGTTCGTTTC	TTAATGCTGCGGAAGTCA
*sept8b*	CTATCGTGGACTACATTGA	ATGAAGTACAGGCAGATG
*pdca*	TGCCGATGTGGAATAATCAGA	ACAGCGTCATTACTCATTCTATCT
*prom1b*	CAGTTGGAGTGACAGTTG	TCAGGTCTCTTATGTTGGT
*18 s*	GAACGCCACTTGTCCCTCTA	GTTGGTGGAGCGATTTGTCT

We performed Gene Ontology (GO) and Kegg Pathway analyses of transcripts enriched in the GFP+ population for genes with log2FC > 0 and FDR < 0.1 (Supplementary File S3). Biological Processes overrepresented in GFP+ populations (all reported here with p < 0.01) include categories that are consistent with a microglia/macrophage identity (for example, (innate) immune response, antigen processing and presentation, microglia differentiation, phagocytosis, and respiratory burst). Interestingly, Biological Process categories overrepresented in GFP+ cells also include wound healing and categories representing cellular motility and mobility (for example, Arp2/3 complex-mediated actin nucleation, macrophage chemotaxis, regulation of cell migration, positive regulation of actin filament polymerization). Cellular Component categories reflect the intracellular compartments required for microglia and macrophage-mediated digestion of engulfed material (for example, vacuole, vacuolar membrane, lysosome, lysosomal membrane, endocytic vesicle, early endosome, late endosome membrane, autophagosome, vesicle, V-type ATPAse categories) and the location of many innate immune receptors involved in initiating immune responses (for example, cytoplasm, NLRP3 inflammasome complex, AIM2 inflammasome complex, extrinsic component of (cytoplasmic side of plasma) membrane). Molecular Function categories reflect the myriad of enzymes involved in degradation of engulfed material in various cellular compartments (for example, hydrolase activity, alpha-mannosidase activity, ribonuclease T2 activity, catalytic activity acting on a protein, epoxide hydrolase activity), and chemokine activity. Kegg pathway analysis again reflected phagocytosis and digestion of engulfed material (for example, lysosome, phagosome, endocytosis), several innate immune receptors (for example, NOD-like receptor signaling pathway, Toll-like receptor signaling pathway, RIG-I-like receptor signaling pathway), and cytokine signaling (Cytokine-cytokine receptor interaction). In addition, Kegg analysis reveals enrichment in MAPK signaling pathway and Metabolic pathways.

### Identification of candidate regeneration-associated transcripts in *mpeg1*:GFP+ cells

One goal of our transcriptome analysis was to reveal transcriptional programs in microglia/macrophages that may be key to successful retinal regeneration. Since we were unable to sort GFP+ cells from undamaged (including saline injected) retinas due to the inability to collect sufficient numbers of GFP+ cells from the cell sorter^38^, we qualitatively compared our list of transcripts enriched in GFP+ retinal cells (by at least log2FC > 2) during regeneration to transcripts identified by Oosterhof *et al*. to be enriched in steady-state zebrafish brain microglia (also by at least log2FC > 2) using the same *mpeg1*:GFP transgenic zebrafish and GFP−based sorting strategy^12^. This comparison was used to generate a collection of candidate genes with putatively enriched expression by microglia/macrophages in a regenerative environment. We used Ensembl IDs as a proxy to determine GFP+ enriched transcripts (with log2FC > 2) in both this study and that reported by Oosterhof *et al*., only found to be enriched in *mpeg1*:GFP+ cells obtained from undamaged brains (“steady-state brain microglia”^12^), or only found to be enriched in *mpeg1*:GFP+ obtained from regenerating retinas (this study).

**Figure 5:**
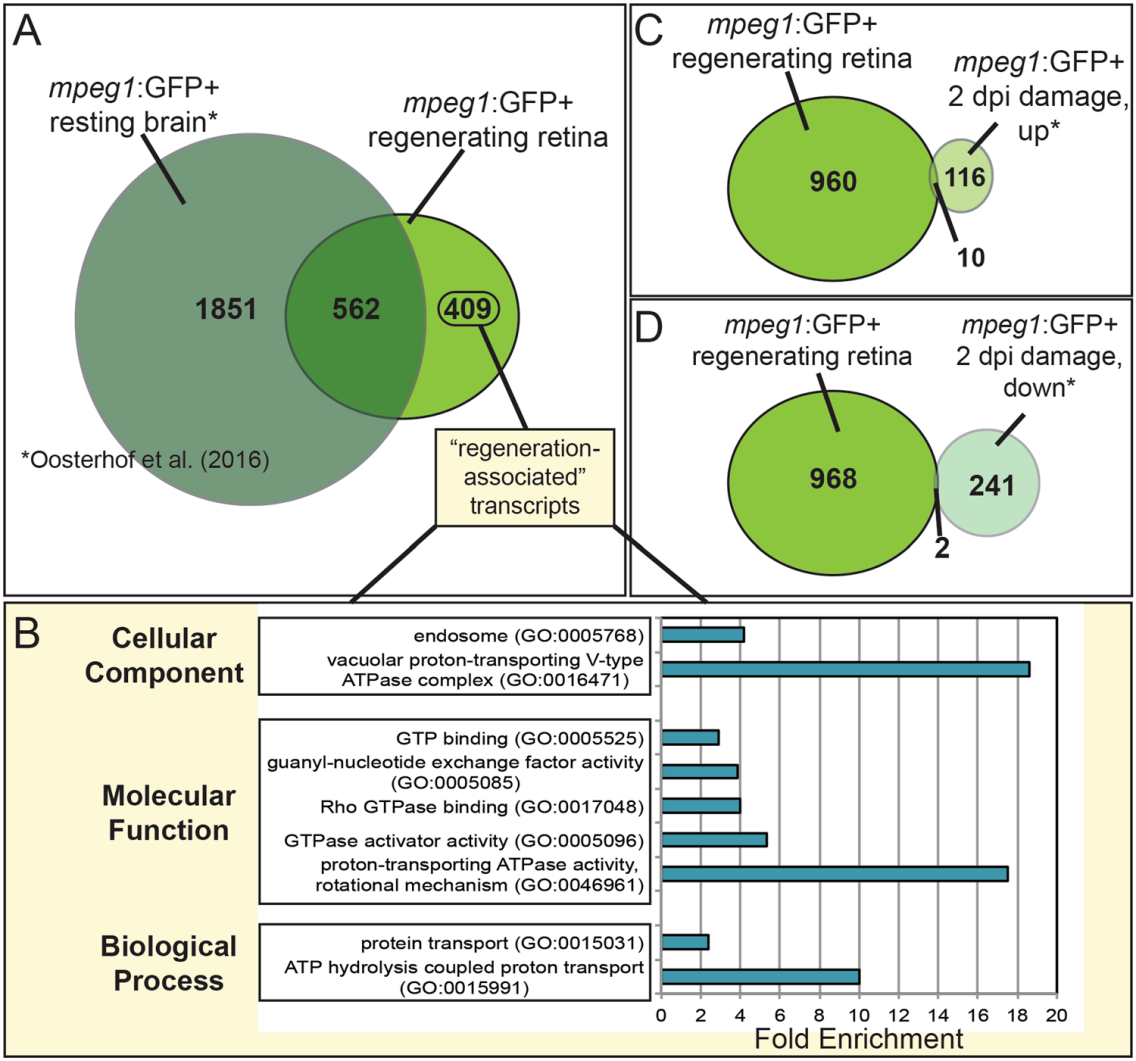
Identification of unique transcripts expressed by *mpeg1*:GFP+ cells during retinal regeneration. (**A**) The list of transcripts found to be enriched in *mpeg1*:GFP+ cells (log2FC > 2) during retinal regeneration (*mpeg1*:GFP+ regenerating retina, this study) was compared to that published to be enriched in *mpeg1*:GFP+ cells (log2FC > 2) obtained from steady-state (*mpeg1*:GFP+ resting brain, Oosterhof *et al*.^12^). “Regeneration-associated” transcripts (409 transcripts) are those found to be enriched in this study (with log2FC > 2), but not in *mpeg1*:GFP+ cells in steady-state brain (with log2FC > 2). (**B**) GO analysis of the 409 “regeneration-associated” transcripts shows enrichment of indicated categories in Cellular Component, Molecular Function, and Biological Process. P < 0.01 for all categories shown. (**C**) The list of transcripts found to be enriched in *mpeg1*:GFP+ cells in this study (again with log2FC > 2) was also compared to transcripts found to be upregulated or downregulated in *mpeg1*:GFP+ cells isolated from acutely damaged (with log2FC > 2, 2 dpi damage, up or down, (**C**,**D**) zebrafish brain (Oosterhof *et al*.^12^). Venn diagrams show the number of transcripts unique or shared between the two studies. Collectively, these comparisons indicate that microglia/macrophages adopt a unique transcriptional program in the context of retinal regeneration. dpi = days post injury.

We found that 562 of our identified transcripts meeting this cut-off criteria were shared with steady-state brain microglia (Fig. 5A and Supplementary File S4), and Gene Ontology analysis placed the majority of these shared transcripts into categories representing known immune cell functions in pathogen response and phagocytic machinery (not shown) representative of core, signature genes associated with microglia and macrophages. These shared transcripts include several genes previously described in microglia (e.g. *slc7a7*, *p2ry12*, *irf8*^62,63,78^), as well as classes of genes such as granulins (e.g. *grna*, *grn1*), complement components (e.g. *c1qa*, *c1qb*, *c1qc*), cytokines (e.g. *il1b*, *tnfb*), and members of the TNF superfamily (e.g. *tnfsf12*, *tnfaip8l2b*, *tradd*). In addition, several novel transcripts identified by Oosterhof *et al*. in zebrafish brain microglia^12^ were also common with our list of transcripts enriched in microglia/macrophages during retinal regeneration (e.g. *tmem104*, *rgs18*, *slc43a3b*, *plaua*).

In terms of transcripts found to be enriched with (cut-off of log2FC > 2) in microglia/macrophages in steady state^12^ versus those found to be enriched in regenerative contexts (this study), our comparison revealed 1851 transcripts unique to *mpeg1*:GFP+ cells in the resting brain and 409 transcripts unique to *mpeg1*:GFP+ cells in regenerating retinas (Fig. 1A and Supplementary File S4, the latter now referred to as “regeneration-associated” transcripts), consistent with a significant shift in the transcriptional program of *mpeg1* expressing cells in the context of active neuronal regeneration. The reduced number of “regeneration-associated” transcripts compared to those from steady state brain could indicate a transcriptional program that is dedicated, at least transiently, to specialized functions in this unique biological context. In support of this idea, transcripts corresponding to toll-like receptor (*tlr*) genes, which comprise a core set of genes involved in detection and initiation of immune response to microbes^79^, showed differential presence in steady-state *mpeg1*-expressing cells (17 *tlr* transcripts) compared to regeneration-associated transcripts (no *tlr* transcripts), with only one *tlr* transcript common between the two states (*tlr1*, Supplementary File S4). This could indicate a shift in dedication of microglia/macrophage function away from immune surveillance and towards other function(s) more appropriate for and dedicated to retinal regeneration at this timepoint. Interestingly, in contrast, upregulation of *tlr* gene expression is characteristic of microglia (and other glia) in several human neurodegenerative diseases^9^.

To suggest alternative functions of microglia/macrophages during retinal regeneration compared to those in “steady-state” tissue, we performed GO analysis on the list of “regeneration-associated” transcripts, which revealed overrepresentation of categories including ATP hydrolysis coupled transport and protein transport (Biological Process, Molecular Funciton), categories related to GTPase activity and reglation (Molecular Function) and vacuolar V-type ATPase complex and endosome (Cellular Component) (Fig. 5B and Supplementary File S5). Together, these categories indicate that microglia/macrophages may be engaged in functions dedicated to active intracellular vesicle transport/fusion (perhaps both endocytic and exocytic) and vacuolar acidification of membrane bound compartments within the cell during retinal regeneration. Collectively, this suggests a phagocytic and/or exocytic function of microglia/macrophages during retinal regeneration. It is possible that neuronal debris from the initial insult remain at this timepoint; however, the 7dpi sampling time is several days later than the peak of neuronal cell death seen at ~1 dpi^18,31^, and axon outgrowth from regenerating ganglion cells is detectable by 7 dpi^18^. Therefore, such a function may instead be involved with maintaining proper tissue conditions and integrity, and/or possibly release of secreted factors, although putative targets of phagocytosis and identity of such secreted factors remains an open-ended question.

Further comparisons of our list of GFP+ enriched transcripts during retinal regeneration to transcripts found by Oosterhof *et al*. to be upregulated, or downregulated, in *mpeg1*:GFP+ cells upon acute brain damage (again with log2FC > 2) revealed only 10, or 2, shared transcripts, respectively (Fig. 5C,D and Supplementary File S6). This suggests that *mpeg1* expressing cells present during active retinal regeneration possess a transcriptome that is substantially different than that of *mpeg1*+ cells responding to acute neuronal damage. This also further supports that the list of candidate “regeneration-associated” transcripts identified from the comparison to steady-state brain *mpeg1*:GFP+ cells (discussed above) represents a transcriptional program expressed by microglia/macrophages in a regenerative tissue state.

## Discussion

Our analysis of regenerating retinal tissue following a tissue disrupting ouabain induced lesion (sparing photoreceptors) reveals that the inner nuclear layer of regenerating retinas contains *mpeg1*-expressing GFP+ immune cells, indicating that these cells identify as microglia/macrophages. These *mpeg1*+ cells appear ameboid in morphologies that often conform to the network of reactive Müller glia. Rather than showing regular distribution throughout retinal tissue, *mpeg1*+ cells are irregularly dispersed and mainly localize to basal regions of regenerating retinal tissues, possibly excluded from regions of MG-derived neuronal progenitor proliferation that occurs when MG-derived progenitors migrate apically towards the outer nuclear layer^19,52,80^. This distribution indicates that localization of *mpeg1*+ cells within regenerating retinal tissue could be spatially regulated. From our analysis of cells expressing PCNA in regenerating retinal tissue at 7 dpi ouabain lesion, most of the PCNA signal can be attributed to reactive MG and MG-derived progenitors, indicating that most cell proliferation at this timepoint is to ultimately regenerate retinal neurons that were destroyed by the lesion. Based on PCNA staining alone, we cannot exclude that at least some *mpeg1*+ cells are also proliferative, since PCNA signal was occasionally associated with both L-plastin and GS. Differentially expressed genes (based on our cut-off criteria) did not show enrichment in GO categories associated with cell division for *mpeg1*+ cells; however, the GFP− population contains proliferative MG and progenitors, therefore differential gene expression analysis would not reveal such transcripts.

In regenerating retinas, the density of immune cells is reduced with distance from the optic nerve head. The reason for this spatial distribution remains unknown. Interestingly, a recent report documented repopulation of homeostatic microglia in a mouse model system revealing a pattern of microglial repopulation that started in central retina and then spread peripherally^81^. However, any repopulation of zebrafish microglia has yet to be demonstrated. In addition, several genes found to be enriched in our study, but not in steady-state microglia^12^ (Fig. 5), may represent orthologues to mammalian genes that may be involved in monocyte to macrophage differentiation (*mafbb*^82,83^, *runx3*^84–86^, *bhlhe40*^87^, *bhlhe41*^87,88^) or macrophage chemoattraction (*cd4-1*, *il16*^89–91^), suggesting that retinas collected at the timepoint in this study may contain other macrophage populations. We are unable to distinguish between CNS microglia and other macrophages based on current markers in zebrafish, so other experimental approaches will need to be employed to determine if either of these are the case.

In addition, the dense network of immune cells at the optic nerve head could represent locations of microglia/macrophage function, such as phagocytosis of debris. Although markers of cell death are only minimally detected at 7 dpi following inner retinal ouabain-induced lesion^18^, cellular and/or tissue debris may remain. By 7 dpi, ganglion cell-associated gene expression has mostly recovered^21^ and ganglion cell markers indicate axon outgrowth occurs by this timepoint^18^. Microglia/macrophages could be involved in functions related to ganglion cell axon regeneration and re-connection to the onh, which are likely required to restore functional vision. However, the time at which axons reach the optic nerve head has not been determined and has been documented only at 21 dpi^21^. Further, microglia have been shown to prune ganglion cell axons during development in mice^2^, and microglia/macrophages are activated within the vicinity of damage following injury to ganglion cell axons in other systems^92–94^, although their functions in this context remain unclear^95^. Further work towards understanding microglial function during regeneration will in turn accelerate progress toward applying regenerative strategies to repair damaged or diseased human retina, including reestablishment of ganglion cell axons^96–98^.

To our knowledge, this is the first work to use RNA sequencing to probe the transcriptome of microglia/macrophages isolated from CNS tissue engaged in a regenerative state. We provide here a list of hundreds of genes enriched in *mpeg1*-expressing cells obtained from highly pure cell populations at a key timepoint during active retinal regeneration in zebrafish. We provide strong evidence that this list of transcripts indeed represents the indicated populations in the desired context, although the *mpeg1*+ may be composed of heterogeneous macrophage cell types (e.g. microglia along with macrophages originating from other sources and/or in distinct phenotypic states). Overall, this dataset provides a novel and exciting resource to the scientific community that will facilitate discovery of microglia/macrophage-specific factors that are crucial to the identity and specification of these immune cell populations (thus potentially leading to identification of better microglia and/or macrophage specific markers) and importantly, identification of factors that function during retinal (and possibly more globally, CNS) regeneration. This dataset will also help to advance our understanding of microglia and macrophage populations in a variety of model organisms.

Our dataset of *mpeg1*:GFP+ enriched transcripts likely represents genes that specify/identify microglia/macrophage cell lineage and may also include genes that have functional roles during regeneration. Therefore, it is important to identify which of these transcripts are specific to the context of retinal regeneration. Due to the inability to sort sufficient numbers of GFP+ cells from control (undamaged or saline injected) retinas with the number of pooled retinas used in this study^38^, we compared our dataset to Oosterhof *et al*. (brain microglia^12^, discussed in Results above). Using this strategy, we identified several hundred genes that may be enriched in microglia/macrophages during active regeneration. It is also worth restating that this list was generated using a cut-off of log2FC > 2, therefore the list of candidate “regeneration associated” transcripts likely includes transcripts that are not expressed in microglia in steady-state as well as those that are putatively upregulated in microglia/macrophages in a regenerative context. Although differences in sequencing methods, the identity of the GFP− population, and analysis methods are a caveat of such a comparison across studies, the high number of genes found to be unique to our study, and changes in categories of genes such as those coding for toll-like receptors and v-ATPase subunits, indicates that the transcriptome of *mpeg1*+ cells in a regenerative tissue environment is significantly different than that of steady-state microglia, and of microglia/macrophages responding to acute neuronal damage. Therefore, this set of “regeneration-associated” transcripts is likely to yield a wealth of opportunity for probing individual candidate genes, and/or cellular pathways, for microglia and/or macrophage-specific functions during retinal (and CNS) regeneration.

Indeed, GO analysis suggests that *mpeg1* expressing cells in contexts of active retinal regeneration have specific functions that may involve vacuolar ATPase-coupled transport related to phagocytosis of extracellular substrates. In support of this, live imaging of microglial phagocytosis during zebrafish brain development indicates that v-type ATPases are involved with intracellular vesicle fusion after engulfment of apoptotic neurons^11^ and a recent report indicates that microglia sense and ingest stressed neurons^99^. Although it remains to be determined, this dedicated function could be required to clean up cellular corpses and/or debris that arise during Müller glia-mediated neuronal regeneration, thus maintaining a tissue microenvironment that is supportive of regeneration. However, levels of cell death at 7 dpi are minimal compared to the initial lesion^18^.

It is worth noting that the extent to which zebrafish brain and retinal microglia in any state are transcriptionally similar or different is not known. Therefore, some of these “regeneration-associated” genes could represent differences between these two types of CNS resident microglia (which would provide a starting point for such a comparison), in addition to regeneration-specific transcriptional programs. Thus, any “regeneration associated” candidate genes will need to be carefully analyzed in control conditions in any follow-up studies while also noting that the presence of transcript does not always equate protein expression. It is also worth noting that the nature of differential gene expression analysis may not reveal any transcripts that are equally or non-differentially expressed in both GFP+ and GFP− populations.

Our list of “regeneration-associated” transcripts may also provide a resource to probe similarities and differences between fish and mammalian microglia responding to neuronal degeneration or in contexts of gliosis. This could ultimately assist us in identifying key similarities and differences in immune cell-mediated factors that may impact the outcome of CNS/retinal regeneration. Such analysis will require careful interpretation of orthology between species as such comparisons may indicate orthologous genes based on protein family similarities that may or may not represent precisely shared functions, and the naming/numbering system in fish does not align with that used in mammals. For example, several chemokines and chemokine receptors were enriched in zebrafish *mpeg1*:GFP+ cells in both the Oosterhof *et al*.^12^ and our study. Chemokines and chemokine receptors are included in rapidly evolving gene clusters and have undergone additional diversification in zebrafish^100,101^. The use of tools to look for orthologues of these chemokine genes in mammals may not provide findings that are simple to interpret, since these chemokines/chemokine receptors may or may not represent well-characterized, and similarly numbered, chemokine genes in mouse or human.

This work has increased our knowledge of the gene expression profile of zebrafish *mpeg1*+ retinal cells isolated from tissue engaged in regeneration. Since Müller glia, Müller glia-derived progenitors and neurons, and microglia/macrophages appear to be the major, and possibly only, cell types present in tissue regions undergoing regeneration following retinal damage, it is essential to understand the contributions of microglia and macrophages to this process. The associated microglia/macrophage specific transcriptome presented here provides insight to a wealth of candidate genes and cellular pathways towards understanding such contributions and to allow comparative work in other model organisms. This foundational information will allow us to begin work to identify the function of microglia/macrophages during successful retinal/CNS regeneration.

## Methods

### Animals

Procedures using zebrafish were performed in compliance with protocols approved by the University of Idaho Animal Care and Use Committee (IACUC). Zebrafish (*Danio rerio*) were maintained on a 14:10 light:dark cycle in 28.5 °C recirculating, monitored system water, housed and propagated according to^102^. The transgenic zebrafish line *mpeg1:GFP* (*gl22 Tg*, GFP expressed in microglia/macrophages^13,49^) used in these experiments was obtained from the Zebrafish International Resource Center (ZIRC). Fish used in these experiments were of both sexes, age 10–12 months.

### Retinal Lesion

Chemical lesioning of zebrafish retinas was performed by intravitreal injection of ouabain (estimated final concentration of 2 µM) in order to destroy inner retinal neurons and spare photoreceptors and Müller glia as reported in^18,21,31,48^. Briefly, a working stock of 40 µM ouabain (ouabain octahydrate, Sigma-Aldrich) was prepared in 0.65% sterile saline (NaCl) solution. Fish were anesthetized by immersion in tricaine solution and an incision was made across the cornea using a sapphire blade. A Hamilton syringe was inserted into the incision, guided behind the lens, and 0.4–0.6 µL of 40 µM ouabain solution was injected into the vitreal chamber. Volume injected was based on diameter of the eye (measured with calipers) and upon calculations based on geometry and volumes of the eye and lens, resulting in an estimated final intraocular concentration of 2 µM^18,103^. Lesions were unilateral; only the right eye was injected. During the procedure, fish were continuously flushed with tricaine solution. Immediately following the procedure, fish were returned to tanks with fresh system water. The right eyes of a separate group of fish were injected with 0.65% sterile saline (NaCl) solution, to serve as controls (same solution was used for saline injections and for preparation of ouabain solution for injection).

For the RNA sequencing experiment, 32 fish were injected in the right eye and upon tissue collection, eight retinas were pooled per sample to create four biological replicates for FACS sorting^38^. To obtain RNA for qRT-PCR confirmation of selected transcripts, 15 fish were injected in the right eye and upon tissue collection, five retinas were pooled/sample to create three biological replicates for FACS sorting. For collection of bulk RNA and tissue processing for retinal cryosections, five control (saline injected) and five ouabain injected fish were used for subsequent analyses.

### Tissue Collection and Processing for Retinal Cryosections

To prepare retinal cryosections, whole eyes were enucleated using fine forceps, transferred to PBS, and the lenses were removed. Eyes were then fixed in phosphate-buffered, 4% paraformaldehyde containing 5% sucrose for 1 hr at room temperature, washed in phosphate-buffered (pH = 7.4) 5% sucrose, and then washed in a graded series ending in 20% sucrose. The following day, tissues were embedded in blocks of a 1:2 solution of OCT embedding medium (Sakura Finetek) and phosphate-buffered, 20% sucrose, and frozen in isobutane, supercooled with liquid N_2_. After freezing solid, tissues were sectioned at 5 micron thickness using a Leica CM3050 cryostat. After overnight desiccation, tissue sections on glass slides were stored at −20 °C until use.

### Immunofluorescence

To stain retinal cryosections, tissue sections (5 micron thickness) were blocked in 20% goat serum for 30 minutes at room temperature, incubated in primary antibody overnight, washed in PBST for at least 30 minutes, incubated in secondary antibody for 1 hour, and washed in PBST for at least 30 minutes. Slides were then mounted in Vectashield + DAPI (Vector Labs), covered with a coverslip, and sealed with clear nail polish. Primary antibodies and dilutions used: rabbit polyclonal anti-zebrafish L-plastin^50,51^ (1:10,000, a kind gift of Dr. Michael Redd), rat anti-PCNA (16D10, 1:200, Chromotek), mouse anti-Glutamine Synthetase (1:1000, BD Transduction Laboratories). Secondary antibodies conjugated to Cy3 or Alexa-Fluor647 (Jackson ImmunoResearch) were used at 1:200 dilution.

### Microscopy and Image Acquisition

Imaging of fluorescently stained retinal sections was performed with a Nikon Andor spinning disk confocal microscope equipped with a Zyla sCMOS camera running Nikon Elements software. Imaging was performed using a 40X (oil immersion) objective. For stitched images of entire retinal cryosections, images were acquired at 40X magnification using the large stitched images feature in Nikon Elements software and stitched based on DAPI signal. Image processing and analysis was performed using FIJI (ImageJ). To quantify pixel intensity of L-plastin+ signal, a continuous line was drawn, with width covering retinal layers, originating at the optic nerve head (onh) and extending to the peripheral retinal tissue. The “plot profile” feature was used to create a plot of pixel intensity (corresponding to L-plastin+ signal) as a function of distance along the line, originating from the onh.

### FACS Sorting of GFP+ and GFP− Populations From Regenerating Retinas

The protocol used for retinal tissue dissociation at 7 days-post ouabain injection (dpi) and subsequent FACS cell sorting was described in detail in Sun *et al*.^38^. Briefly, following dark adaption followed by retinal tissue dissociation, GFP+ and GFP− populations were isolated by FACS using a SONY Cell Sorter SH800 at Washington State University. The gating strategy for FACS was based on the GFP signal intensity of *mpeg1*+ cells (GFP+ cells), as well as the scatter characteristics (forward scatter, FSC; and side scatter, SSC) of the target cells, and the same gating was used for all biological replicates. A 488 nm laser with 525/50 photomultiplier tube (PMT) was used by the cell sorter to illuminate GFP+ cells for GFP−based sorting.

### RNA Isolation, 3′mRNA-seq Library Synthesis, and RNA Sequencing

RNA was extracted from sorted GFP+ and GFP− populations using NucleoSpin® RNA XS kit (Machery-Nagel) following the manufacturer’s protocol. RNA samples were quantified and quality-checked on a Fragment Analyzer Automated CE System (Advanced Analytical) at the IBEST Genomics Resources Core, University of Idaho. RNA yields (~14 ng/sample for GFP+ and ~29 ng/sample for GFP− populations) and Fragment Analyzer results are reported in^38^. Libraries for sequencing were prepared using the Lexogen QuantSeq. 3′mRNA-Seq Library Prep Kit FWD using 16 cycles of PCR as per manufacturer instructions. The following steps were then performed prior to sequencing: (1) additional (non-method specified) bead cleanup (1:1 Magbio), (2) five additional cycles of PCR with KAPA library amplification kit (ABI), (3) additional (non-method specified) bead cleanup (1:1 Mabgio), (4) Fragment Analyzer QA, (5) pool equimolar amounts, (6) additional (non-method specified) bead cleanup (2 × 1:1 Mabgio), (7) Fragment Analzyer (QA), (8) five additional cycles PCR with KAPA library amplification kit (ABI). The QuantSeq. 3′-mRNA sequencing method is described in^104^.

Reads were sequenced at the University of Oregon on a SE75 run using an Illumina HiSeq. 4000. Reads were demultiplexed by University of Oregon. Approximately 27–41 million reads were obtained per sample library, with the exception of one library, which nevertheless provides depth suitable for downstream analysis towards expression profiling^105,106^, and therefore was included in the analysis to increase statistical power. Reads were quantified using Salmon v0.9.1 using the quasi-mapping-based mode, and using “–noLengthCorrection” setting^107^ against the Ensembl release-90 *Danio rerio* transcriptome (GRCz10). Mapping rates were approximately 61–69% for all samples. Sample reads and mapping rate are summarized in Supplemental Table S1.

### Differential Gene Expression Analysis

Analysis was carried out using methods derived from^108^. Briefly, data was imported into R (R Core Team (2017), https://www.R-project.org/) using tximport^108^. Differential expression analysis was then carried out using DESeq2^109^, and moderated log2FC is reported to normalize for lowly expressed transcripts. Gene clustering ananlysis was performed using the WGCNA strategy and package^110^, and the resulting analysis is summarized in Supplementary File S1.

### Gene Ontology and KEGG Pathway Analysis

GO analysis was performed to identify over-represented GO terms for genes found to be enriched in GFP+ compared GFP− cells with criteria of log2FC> 0 and p < 0.1 using GOstats^111^. KEGG analysis was performed to identify over-represented KEGG pathways for genes found to be enriched in GFP+ versus GFP− cells using the kegga function within the Limma package^112^. Enriched categories shown are based on a cut off of p < 0.01. GO analysis of transcripts relevant to comparisons to Oosterhof *et al*.^12^ was performed using the Gene Ontology Consortium GO Enrichment Analysis Tool (www.geneontology.org).

### Quantitative PCR (qPCR)

cDNA samples intended for quantitative PCR (qPCR) follow-up of RNA-seq hits, quality of purified RNA was analyzed using a Nanodrop spectrophotometer. cDNA was synthesized using Superscript® III First-Strand Synthesis kit (Invitrogen) using random hexamer primers. qPCR was performed using SYBR-Green PCR Master Mix with amplification run on a model 7900HT Fast Real-Time PCR System (Applied Biosystems, Inc.). Three technical replicates were performed for each biological replicate, and template cDNA was diluted 1:20 prior to addition to the qPCR reaction. Where appropriate, relative quantitation of gene expression between sorted *mpeg1*:GFP+ and GFP− samples was determined using the 2^−ΔΔCt^ method with *18 s* ribosomal gene as the endogenous reference gene. In cases where average Ct was found to be >34 for the samples analyzed, we consider this amplification to be unreliable because Cts in the range of 34–40 indicate transcript quantities that approach single copy level in the sample well, and the qPCR instrument manufacturer recommends against their use in analysis such as ΔΔCt. In several instances, Ct values were returned as “undetermined” by the qPCR instrument (indicating a Ct was not obtained from the sample well by cycle 40) for either the GFP+ or GFP− population. Primer sequences are shown in Table 5.

## Supplementary information

Supplementary File S1

Supplementary File S2

Supplementary File S3

Supplementary File S4

Supplementary File S5

Supplementary File S6

## Data Availability

The RNAseq dataset generated and analyzed in the current study are available in the GEO repository (Accession GSE120467). The other datasets generated and analyzed during the current study are available from the corresponding author on reasonable request.
