# Supplementary material for: Regeneration associated transcriptional signature of retinal microglia and macrophages
Source: Sci Rep. 2019 Mar 18;9:4768. doi: 10.1038/s41598-019-41298-8 (PMC6423051; doi:10.1038/s41598-019-41298-8)
Supplement: Supplementary file 1 — Supplementary File S1 [file 41598_2019_41298_MOESM1_ESM.pdf]

Regeneration associated transcriptional signature of retinal microglia and macrophages

Diana M. Mitchell*, Chi Sun, Samuel S. Hunter, Daniel D. New, Deborah L. Stenkamp

Supplemental Table S1: Reads and genome coverage of sequenced samples.

Sample	Reads	Coverage (Mapping Rate)
GFPpos 1	37,474,108	62.8629%
GFPpos 2	38,871,628	60.9751%
GFPpos 3	27,273,785	64.125%
GFPpos 4	33,796,965	62.6812%
GFPneg 1	39,864,003	69.2867%
GFPneg 2	35,522,883	62.0528%
GFPneg 3	10,817,409	67.6374%
GFPneg 4	41,406,171	66.9648%

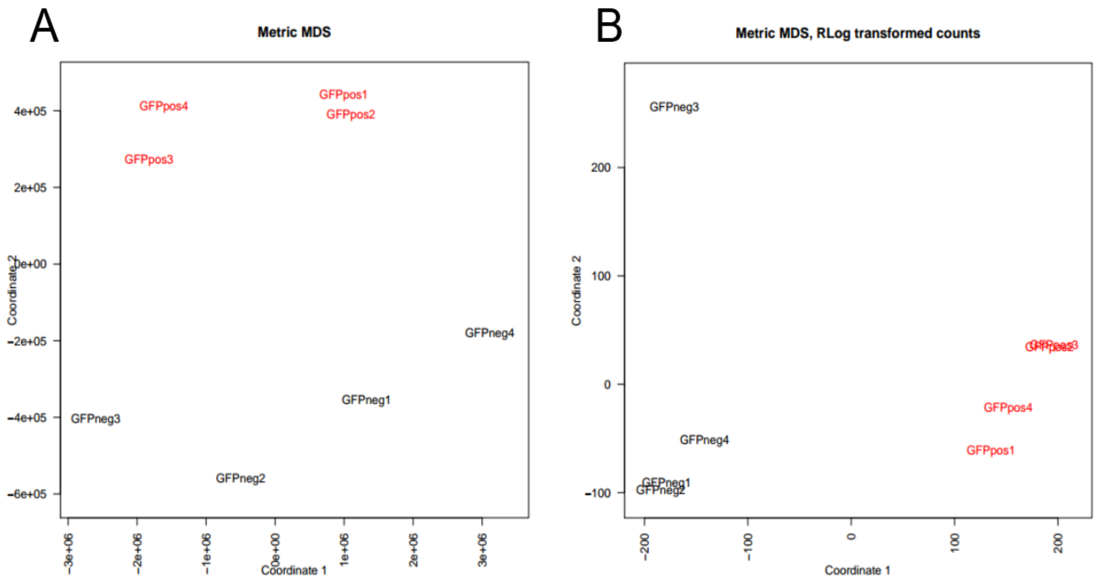

Supplemental Figure S1.
A. We used Metric MDS clustering of Salmon transcript quantification values to confirm that biological replicates showed good clustering by sample type. Samples clustered by type on Coordinate 2, but show some variability within type across Coordinate 1.
B. To determine whether variability within treatment group might be driven by non-biological signals such as differences in sequencing depth, or a small subset of genes with high levels of expression, we applied the regularized log transformation (rlog) to the data and again clustered using Metric MDS. This resulted in tight clustering of the two treatment groups along both Coordinate 1 and 2, except for sample GFPneg3 which showed separation on Coordinate 2. However, because Coordinate 1 explained 54% of the variance in the data, while Coordinate 2 explained only 15% we chose to retain GFPneg3 for further analysis.
